# Sialic acid receptor detection in the human respiratory tract: evidence for widespread distribution of potential binding sites for human and avian influenza viruses

**DOI:** 10.1186/1465-9921-8-73

**Published:** 2007-10-25

**Authors:** John M Nicholls, Anthony J Bourne, Honglin Chen, Yi Guan, JS Malik Peiris

**Affiliations:** 1 Pathology Department, The University of Hong Kong, Pok Fu Lam, Hong Kong, Hong Kong SAR; 2 Pathology Department, Adelaide Women and Children's Hospital, Adelaide, South Australia 5006, Australia; 3 Microbiology Department, The University of Hong Kong, Pok Fu Lam, Hong Kong, Hong Kong SAR

## Abstract

**Background:**

Influenza virus binds to cell receptors via sialic acid (SA) linked glycoproteins. They recognize SA on host cells through their haemagglutinins (H). The distribution of SA on cell surfaces is one determinant of host tropism and understanding its expression on human cells and tissues is important for understanding influenza pathogenesis. The objective of this study therefore was to optimize the detection of α2,3-linked and α2,6-linked SA by lectin histochemistry by investigating the binding of Sambucus nigra agglutinin (SNA) for SAα2,6Gal and Maackia amurensis agglutinin (MAA) for SAα2,3Gal in the respiratory tract of normal adults and children.

**Methods:**

We used fluorescent and biotinylated SNA and MAA from different suppliers on archived and prospectively collected biopsy and autopsy specimens from the nasopharynx, trachea, bronchus and lungs of fetuses, infants and adults. We compared different methods of unmasking for tissue sections to determine if these would affect lectin binding. Using serial sections we then compared the lectin binding of MAA from different suppliers.

**Results:**

We found that unmasking using microwave treatment in citrate buffer produced increased lectin binding to the ciliated and glandular epithelium of the respiratory tract. In addition we found that there were differences in tissue distribution of the α2,3 linked SA when 2 different isoforms of MAA (MAA1 and MAA2) lectin were used. MAA1 had widespread binding throughout the upper and lower respiratory tract and showed more binding to the respiratory epithelium of children than in adults. By comparison, MAA2 binding was mainly restricted to the alveolar epithelial cells of the lung with weak binding to goblet cells. SNA binding was detected in bronchial and alveolar epithelial cells and binding of this lectin was stronger to the paediatric epithelium compared to adult epithelium. Furthermore, the MAA lectins from 2 suppliers (Roche and EY Labs) tended to only bind in a pattern similar to MAA1 (Vector Labs) and produced a different binding pattern to MAA2 from Vector Labs.

**Conclusion:**

The lectin binding pattern of MAA may vary depending on the supplier and the different isoforms of MAA show a different tissue distribution in the respiratory tract. This finding is important if conclusions about the potential binding sites of SAα2,3 binding viruses, such as influenza or human parainfluenza are to be made.

## Background

There are two main epithelial cell types in the bronchus – ciliated cells and goblet cells that secrete mucus. Within the submucosa there are also submucous glands present. The goblet cells have a glycoprotein which is acidic due to the presence of sialic sulphate groups and this secretion may vary with various diseases [1]. SAα2,6Gal has been reported to be present on the apical surface of ciliated cells but there have been conflicting reports about SAα2,3Gal expression on cell types. In cultured epithelial cells SAα2,6Gal appears to be present on non-ciliated (goblet) cells while SAα2,3Gal is present on ciliated cells [2]. On the contrary, others have reported that SAα2,3Gal expression is found in goblet cells [3]. In addition, the patterns of glycosylation and the expression profile of SAα2,6 on cell surfaces may change during the course of developmental differentiation and following oncogenesis. For instance, SAα2,6Gal binding is weak during the glandular stage of lung development but increases as the lung matures [4]. Furthermore, if cells are exposed to inflammation and tumour necrosis factor there may be qualitative changes in glycosylation and the glycosyltransferases that lead to sialylation [5]. Recent publications, however, have indicated that both SAα2,3Gal and SAα2,6Gal may be present in the respiratory tract but with different distributions [6,7]. The presence or absence of these SA is important as human influenza A strains have been reported previously to preferentially attach to cells with SAα2,6Gal linkages and avian strains preferentially bind SAα2,3Gal [8].

The affinity of the attachment of the HA to cell surface receptors is believed to be an important determinant in tissue tropism of the virus and constitutes part of the species barrier that keeps avian influenza viruses from readily infecting humans. Pigs contain a respiratory epithelium that has been reported to contain both "avian-virus" binding SAα2,3Gal and "human-virus" binding SAα2,6Gal linkages supposedly explaining why they can be infected with both human and avian influenza viruses [9]. Therefore pigs have been regarded as a hypothetical "mixing" vessel where re-assortment of avian and human viruses can take place, potentially leading to the emergence of pandemic influenza [9]. Given the presumed importance of the affinity of the influenza virus for its receptor, the distribution of SAα2,6Gal and SAα2,3Gal expression in the human respiratory tract is critically important for the understanding of influenza pathogenesis.

Some of the studies on the types of SA expressed on cell surfaces have been done on sialic acids extracted from cell membrane homogenates. However, for the understanding of influenza pathogenesis and pandemic emergence, it is important to have methods that can define the profiles of SAα2,6Gal and SAα2,3Gal in histological tissue sections *in situ*. The SAα2,6Gal and SAα2,3Gal expression on histological specimens *in situ *can be done using fluoresceinated lectins and by histochemistry. Since the early 1990's many histology laboratories have used unmasking or retrieval techniques to enhance immunohistochemical detection of antigens.

In this study we address three issues. The first was to investigate if antigen unmasking or retrieval would affect lectin-ligand expression in histological tissues. The second issue was to compare the findings obtained by using lectin fluorescence with cytochemistry for lectin-ligand analysis in the same tissue samples. Finally, we wanted to use optimized methods to re-evaluate the distribution of the SAα2,6Gal and SAα2,3Gal in human respiratory tissues and then correlate this with the reported presence or absence of influenza infection in different parts of the respiratory tract.

## Methods

Biopsy samples were collected from the archived files of the Histopathology Department of Queen Mary Hospital, Pok Fu Lam, Hong Kong. Five surgically removed lungs from children with congenital cystic adenomatoid malformation (CCAM), seven non-neoplastic bronchial biopsies from patients investigated for possible malignancy, eight normal nasopharyngeal biopsies from patients with suspected nasopharyngeal carcinoma and eight lung biopsy samples from 20–40 week abortuses were also used. Seven biopsy tissues of CCAM, representing paediatric lung tissues of ages 1 month to 7 years were also retrieved from the files of the Department of Histopathology, Adelaide Women's and Children's Hospital, Adelaide, South Australia. All tissues had been fixed in 10% neutral buffered formalin, processed into paraffin and stored at room temperature. Tissue blocks from the lungs of five patients who had died of acute bacterial pneumonia were used as a non-influenza comparison. Intestinal tissue from four ducks kindly provided by the Agriculture, Fisheries and Conservation Department, Government of HKSAR, were used as a positive control for SAα2,3Gal binding. The research was approved by the Ethics Committee of the University of Hong Kong/Hospital Authority Western Cluster.

We used lectin histochemistry and fluorescence which is the standard method of detection of the SA linkages [10]. Lectin analysis was performed using the fluorescein labelled lectins Sambucus nigra agglutinin (SNA) which primarily detects 6-linked sialic acids and Maackia amurensis agglutinin (MAA) which primarily identifies 3-linked sialic acids. Both fluorescein isothiocyanate-labelled (FITC) and tetramethyl rhodamine isothiocyanate-labelled (TRITC) were used as fluorochromes and purchased from EY Laboratories (San Mateo, California). Additional FITC conjugated MAA1 was purchased from Vector Laboratories (Burlingame, CA). When peroxidase or biotin conjugation was used the conjugates were purchased from EY Laboratories (SNA and MAA) and Vector (MAA1 and MAA2). Digoxigenin labelled SNA and MAA was purchased from Roche as part of the Dig-Glycan Detection Kit.

For the initial trial of optimization for oligosaccharide ligand retrieval methods one lung block from a case of CCAM was used. The tissues were sectioned at 5 μm and deparaffinized. Control sections had no retrieval. Two different methods were used for retrieval: microwave and enzyme digestion. For microwaving, an Energy Beam Sciences microwave processor was used together with 2 types of buffer. 0.1 M EDTA and 10 mM citrate buffer was used and the sections were microwaved for 10,15, 20 and 25 minutes at 95°C. Two enzyme digestion methods were used: trypsin and pronase, and for both these methods sections were incubated for 15 mins at 37°C.

Single fluorescent studies were performed as follows. The sections were microwaved in 95°C citrate buffer pH 6.0 for 15 minutes, washed with 0.05 M Tris Buffer Saline (TBS) pH 7.6 and then incubated with either 1/100 FITC conjugated SNA (EY Laboratories), or 1/100 FITC conjugated MAA (EY Laboratories) for 1 hour at room temperature in the dark. Double fluorescent studies were performed according to the method of Mason et al. [11]. Briefly, the sections were microwaved in 95°C citrate buffer pH 6.0 for 15 minutes, washed with 0.05 M Tris Buffer Saline (TBS) pH 7.6 and then incubated with 1/100 TRITC conjugated SNA (EY Laboratories) and 1/100 FITC conjugated MAA(EY Laboratories) for 1 hour at room temperature in the dark. The sections were washed with TBS 3 times for 5 minutes each and the nuclei stained with 5 μg/ml DAPI for 4 minutes followed by three washes with TBS of 5 minutes each and mounting with DAKO fluorescent mount (Dako, Glostrup, Denmark). Fluorescent examination was with a Nikon Eclipse microscope with SPOT Pursuit Camera (Sterling Heights, MI) and Image-Pro Plus software (MediaCybernetics, MD) was used.

Lectin horseradish peroxidase (HRP) detection: the sections were microwaved in 10 mM citrate buffer pH 6.0 for 15 min, blocked with 3% H_2_O_2 _in TBS for 12 min and after washing with TBS 3 times, 5 minutes each were then incubated with 1/50 HRP conjugated SNA (EY Laboratories) and 1/50 HRP conjugated MAA (EY Laboratories) at room temperature for 1 hour respectively. After 3 further washes in TBS the sections were developed with AEC substrate kit (Vector Laboratories) at RT for 30 minutes followed by counterstaining with Mayer's haematoxylin and mounting with DAKO aqueous mount (Dako, Glostrup, Denmark).

Lectin biotin detection: The sections were microwaved in 10 mM citrate buffer pH 6.0 at 95°C for 15 min then blocked with 3% H_2_O_2 _in TBS for 12 min and with avidin/biotin blocking kit (Vector). They were then incubated with 1/100 biotinylated MAA1 (or 1/100 biotinylated MAA2) (Vector) for either 1 hour at RT or 4°C overnight, blocked with 1% bovine serum albumin for 10 mins at RT, and then incubated with strep-ABC complex (Dako Cytomation, K-0377) diluted 1/100 for 30 mins. at room temperature. Development was performed using the AEC substrate kit (Vector) at room temperature for 10 minutes. The nuclei were counterstained with Mayer's hematoxylin and then the sections were dried and mounted with DAKO aqueous mount (Dako Cytomation). Duck intestine sections were used as controls with and without pre-treatment with SAα2,3 specific neuraminidase from Glyko to ensure that sialic acids were specifically targeted. Stain intensity was measured semi-quantitatively using duck MAA as a control. Similar stain intensity to the duck intestine was graded as strong (++) and a weaker pattern as +.

To determine lectin binding profiles we used data from the Consortium for Functional Glycomics (CFG) web site [12] using glycan array data for MAA1(also known as MAL), SNA and a human H5N1 virus, (A/Vietnam/1203/04).

## Results

### Microwave retrieval increased lectin binding

**Figure 1:**
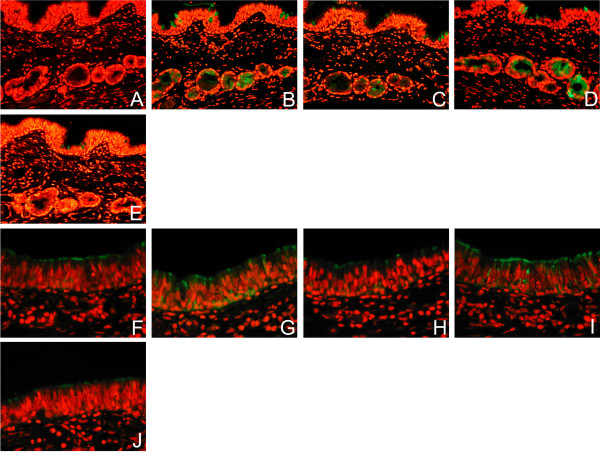
**Effect of different retrieval techniques on lectin binding**. Single labelling of paediatric respiratory mucosa by FITC- conjugated Sambucus nigra agglutinin (SNA) (A-F) and Maackia amurensis agglutinin (MAA) (G-J) using different methods of retrieval. No antigen retrieval, (A) and (F), Citrate buffer (B) and (G), EDTA (C) and (H), Pronase (D) and (I), and Trypsin (E) and (J). Stain intensity was graded as strong (++) and a weaker pattern as +. In the absence of unmasking techniques there was minimal to weak (-/+) SNA binding and weak (+) MAA binding in the basal epithelium and epithelial cells of the bronchial mucosa of paediatric tissues. All forms of retrieval enhanced the lectin staining of the surface epithelial cells and mucus containing cells for both SNA and MAA. Examination with dual FITC/Rhodamine filter. Magnification × 200.

In the absence of unmasking techniques and using the lectins SNA and MAA from EY Labs, there was minimal to weak (-/+) SNA binding and weak (+) MAA binding in the basal epithelium and epithelial cells of the bronchial mucosa of paediatric tissues. All forms of retrieval enhanced the lectin binding to the surface epithelial cells and mucus containing cells for both SNA and MAA (Figure 1) and this appeared to be most prominent in the submucous glands. Trypsin and pronase digestion also produced increased binding, but it also tended to result in more surface epithelial denudation of cells from the section. When microwave heating was used the increase in staining was maximal after 15 minutes. As citrate buffer heating appeared to just as beneficial as other forms of retrieval this was chosen as the preferred method of unmasking for future work. Tissue sections of duck intestine was used as a control and this showed MAA binding on the surface of the columnar cells in keeping with its SAα2,3Gal moiety.

### There is different binding of MAA isoforms to the upper and lower respiratory tract in adult and paediatric tissues

**Figure 2:**
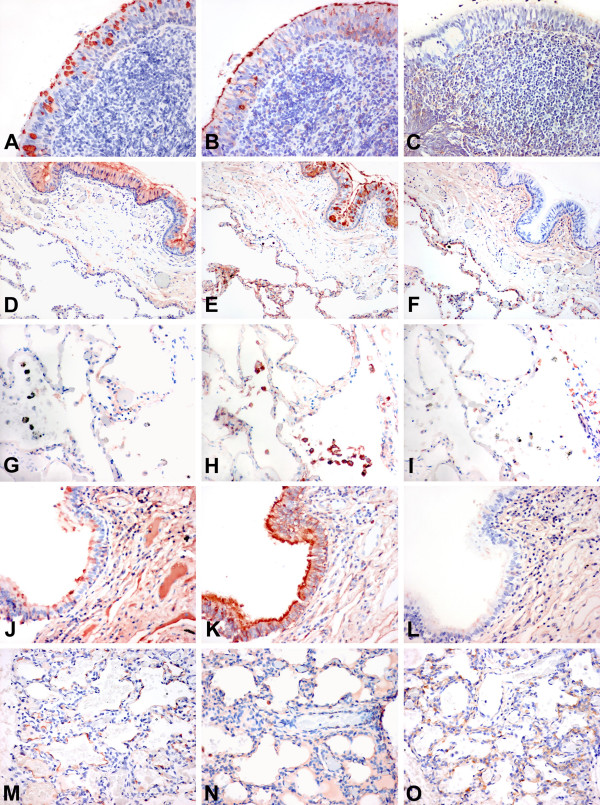
**Lectin binding to upper and lower respiratory tract**. Tissue distribution of Sambucus nigra agglutinin (SNA) for SAα2,6, Maackia amurensis agglutinin 1 (MAA1), and Maackia amurensis agglutinin 2 (MAA2) for SAα2,3 binding in the adult and paediatric respiratory tract. Serial sections of nasopharynx (A-C), adult bronchus (D-F), adult lung (G-I), paediatric bronchus (J-L), and paediatric lung (M-O) are shown and stained with SNA (A,D,G,J,M), MAA1 (B,E,H,K,N) and MAA2 (C,F,I,L,O). The adult nasopharynx shows SNA and MAA1 binding in the epithelium but no MAA2 binding. A similar pattern is also present in the adult bronchus and in addition the pneumocytes show MAA1 and MAA2 binding (E,F). Alveolar macrophages (G-I) demonstrate minimal SNA and no MAA2 binding but are positive for MAA1. The paediatric bronchus shows a greater binding of the epithelium with MAA1 (K) than the adult (E). The pneumocytes (M) also show more SNA binding than the adult (G). Staining using HRP conjugated SNA and biotin conjugated MAA1 and MAA2. (A-F) and (J-L) at 200 × magnification and (G-I) and (M-O) at 400 × magnification.

The 8 samples of fetal tissue at 20 weeks gestation showed strong binding (++) of MAA to the bronchial epithelium and to the developing pneumocytes with absent SNA detected. With advancing development the SNA binding increased and there was a switch in the MAA1 and MAA2 binding between the alveoli and bronchi (Table 1). The paediatric tissues from children with CCAM showed strong (++) binding in all cases of MAA to the bronchial epithelium but not to the alveoli. SNA was also strongly (++) bound to the bronchial epithelium but there was weaker (+) binding to the alveoli. Because we found greater binding of MAA in the respiratory tract than previously reported using the MAA from one supplier (EY Laboratories) we tested the MAA from another supplier (Vector) to verify the results. The MAA from EY labs has been identified as a combination of 2 isoforms of MAA – MAA1 and 2 which though both identifying SAα2,3Gal have different recognition patterns for the inner fragments. While MAA2 is specific towards SAα2,3Galβ1,3GalNAc and has been used to detect the "traditional" avian influenza receptor, MAA1 is more specific towards SAα2,3Galβ1,4GlcNAc [13]. When an analysis of sequential sections from the upper and lower respiratory tract was performed a number of consistent findings were observed. Firstly, SNA binding was more widespread in the upper than the lower respiratory tract and this was more pronounced in the adult tissues (Fig 2A,D,G,J,M). It was also present in the mucus secreting cells as well as the ciliated cells with a strong intensity (++). In the adult lung the pneumocytes showed only weak binding (+) of SNA (Fig 2G) but there appeared to be stronger binding (++) to the epithelium of the lungs of children (Fig 2M). Secondly, MAA1 was widely bound throughout the all the respiratory tract of children and adults and did not vary with age after delivery (Fig 2B,E,H,K,N). It bound to ciliated cells as well as the mucus secreting cells. In the paediatric bronchus it also bound to the basal cells (Fig 2K). Thirdly, MAA 2 binding appeared to be limited only to pneumocytes (+ – ++) and did not bind to cells in the nasopharynx or the bronchial epithelium (Fig 2F,O).

**Table 1:** Summary of lectin binding intensities in fetal, paediatric and adult respiratory tissues using Sambucus nigra agglutinin (SNA) and Maackia amurensis 1 and 2 (MAA1 and MAA2).

	**Fetus (n = 8)**		**Paediatric lung (n = 12)**			**Adult NP (n = 8)**		**Adult bronchus (n = 7)**		**Adult lung (n = 5)**		
	
	Alv	Bronch	Alv	Bronch	Gland	Epith	Gland	Epith	Gland	Macro	Pneu	Bronch
SNA	- → +	- → +	+	+/-	+	++	++	++	++	+	+	+
MAA1	++ → -	- → +	-	++	+	++	++	++	++	+/++	-	++
MAA2	- → +	+ → -	++	-	+/-	-	-	-	-	-	++	-

Alv = alveolus, Bronch = bronchial epithelium, Epith = epithelium, Macro = macrophage and Pneu = pneumocyte. The arrows in fetal tissue indicate the changes in expression from 20 weeks gestation to term.

### Within the adult bronchus there was heterogeneous distribution of lectin binding

**Figure 3:**
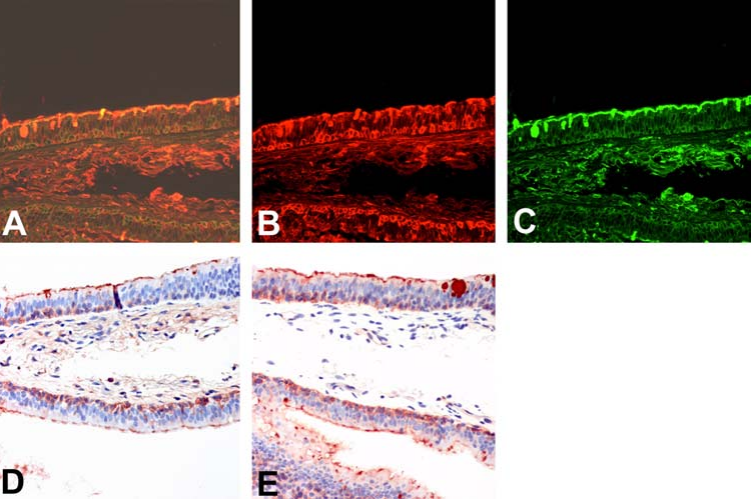
**Lectin binding to bronchial epithelium using different detection methods**. Serial sections for comparison of detection methods for lectin binding of Sambucus nigra agglutinin (SNA) to SAα2,6 and Maackia amurensis (MAA) agglutinin for binding to SAα2,3 in adult bronchial epithelium. Double fluoresecence for FITC labelled SNA and TRITC labelled MAA shows a heterogeneous pattern (A), with more binding to the basal epithelium with MAA (B) than SNA (C). HRP labelling of MAA (D) and SNA (E) shows a similar pattern of binding to the fluorescent labelled lectin. 200 × magnification.

When dual labelling was performed on the bronchial tissues of adults and of children using SNA and MAA, we identified a heterogeneous distribution of SNA and MAA binding in the epithelium with no clear distinction between ciliated and non-ciliated cells resulting in a mixed and occasionally dual expression of both SAα2,3Gal as well as SAα2,6Gal in the ciliated cells, goblet cells and basal cells (Fig 3A). We also found a similar staining pattern when non-fluorochromes were used (Fig 3B–E).

### The MAA from different suppliers has different binding patterns

**Figure 4:**
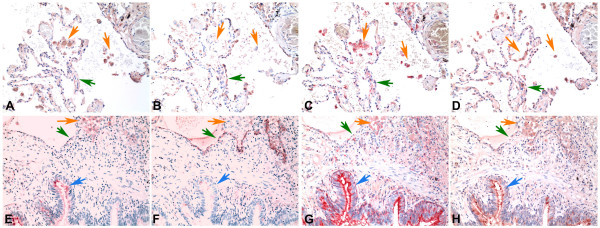
**Comparison of MAA binding using MAA from different suppliers**. Serial sections of adult lung tissue for comparison of lectin binding of Maackia amurensis (MAA) for SAα2,3Gal. Biotin conjugated MAA1(also known as MAL) from Vector Laboratories (A) and (E), Biotin conjugated MAA2 (also known as MAH) from Vector Laboratories (B) and (F), Digoxigenin conjugated MAA from Roche (C) and (G) and HRP conjugated MAA from EY Laboratories (D) and (H). Orange arrows indicate alveolar macrophages, Green arrows indicate alveolar pneumocytes and blue arrows indicate bronchiolar epithelium. Haematoxylin counterstain 200 × magnification.

Because we found that there was a different binding pattern in the upper and lower respiratory tract for MAA1 and MAA2 using these lectins from Vector labs we then investigated whether the MAA from 2 other suppliers – EY Laboratories and Roche would identify one or both of the MAA isoforms. We therefore used serial sections from adult lung tissue and stained them using the Vector MAA1 and MAA2, digoxigenin labelled MAA (Roche) and biotinylated MAA from EY Laboratories. While EY Laboratories state that their MAA is a combination of 2 isoforms, this information is not evident from Roche. As expected using MAA1 (Fig 4A,E) the alveolar macrophages (orange arrows) and bronchial cells (blue arrow) bound MAA but pneumocytes (green arrow) showed no binding. Conversely MAA2 (Fig 4B,F) bound to pneumocytes (green arrows). The digoxigenin labelled MAA from Roche (Fig 4C,G) mainly bound to macrophages (orange arrows) and bronchial cells (blue arrow) but there was minimal binding to pneumocytes (green arrows) indicating that this MAA is mainly identifying MAA1 rather than MAA2. The MAA from EY Laboratories (Fig 4D,H) showed a weak (+) binding to the pneumocytes (green arrows) as well as strong binding (++) to bronchial epithelial cells and macrophages but it should be noted that this binding to pneumocytes appeared weaker than MAA2.

Analysis of the glycan array binding data of MAA1(also known as MAL), SNA and H5N1 submitted to the CFG showed that MAL did detect SAα2,3Galβ1,4GlcNac(glycan numbers 235–238) with detection of the sulphated forms (216 and 227) [see Additional file 1]. There was absent binding to SAα2,3Galβ1,3GalNAc (number 202). If a reading of 2000–4000 is taken as a weak binding then MAL also had weak binding to SAα2,6Galβ1,4Glc (number 249) but not when the Glc was replaced by GlcNAc (numbers 245,246). As expected, the H5N1 (Viet04) virus bound strongly to SAα2,3Gal termini and many of the strong virus- binding oligosaccharides were identified by MAA1 (e.g. numbers 235–237). While MAA2 or (MAH) has not been analysed in glycan arrays, it preferentially detects the O-linked SAα2,3Galβ1,3 [SAα2,6]GalNAc [13]. This glycan (221) shows strong binding for H5N1 but minimal binding for MAA1 [see Additional file 1]. MAA1 does show high affinity to non-sialic acid residues especially those with a sulphate moiety (e.g. glycan numbers 28–38) which has led to it not being recommended for use for identifying the SAα2,3 terminated oligosaccharides and indeed we found that using neuraminidase treatment of sections that MAA1 and 2 binding could be abolished in duck intestine tissues but trials in human tissues did show residual MAA1 binding after treatment (data not shown).

## Discussion

Cells of the respiratory tract express a number of glycan containing conjugates on the cell surface, many of which terminate with *N-*acetylneuraminic (sialic, SA) acids – a series of 9-carbon sugars. Influenza virus infection of humans involves binding of the virus haemagglutinin (HA) to these sialyoligosaccharides on the surface of cells of the respiratory tract. In addition, the virus neuraminidase (NA) cleaves the sialic acid on the host cell and is important in releasing newly formed virus from the cell after virus replication is completed so these virions can spread out in search of other cells to infect. Since respiratory mucus is also rich in SA, this provides a potential barrier to the spread of newly formed virions. By cleaving these SA, the influenza virus NA facilitates the virus spread through this mucus layer [14]. Thus the balance between the affinity of binding between the virus and the cell receptor and the virus-releasing activity of the NA is critical to virus replication in a host species.

The crystal structures of HA shows that the terminal sialic acids bind in a groove at the top of the HA molecule [15]. Previous investigations had indicated that avian viruses would preferentially bind SAα2,3Gal and human viruses SAα2,6Gal [8]. The avian and human H5N1 viruses causing the "bird flu" outbreak in Hong Kong in 1997 had affinity for binding to SAα2,3Gal but the virus associated with human disease in Hong Kong in 2003 had affinity to bind to both avian-like SAα2,3Gal and human-like SAα2,6Gal [16]. H5N1 disease in humans has been reported to be different from conventional human influenza viruses (H3N2 or H1N1) in that the lower (rather than upper) respiratory tract is the major target for virus replication [7].

Using retrieval methods and selection of lectin conjugate we have demonstrated that the lectin binding to the SAα2,3Gal receptor for avian influenza viruses is more widely expressed in the respiratory tract of humans than previously documented [7]. In particular, we demonstrated that unlike the previous reports that indicated certain type of cells had only one lectin binding profile, SAα2,6 and SAα2,3Gal was found in ciliated epithelium, goblet cells and submucous glands in the bronchus as well as pneumocytes of the alveoli, and SAα2,3 was also present in the metaplastic epithelium. In keeping with an earlier report [4], we found that neonatal pneumocytes expressed mainly SAα2,3Gal and the neonatal bronchus was also primarily SAα2,3Gal expressing. The respiratory tract of young children showed mainly SAα2,3Gal with a lower level of SAα2,6Gal expression than adult tissues. This may, in part explain why children appear to be more susceptible to avian influenza H5N1 in the recent outbreaks in East Asia.

The difference between our studies and previous ones on sialic acid expression in respiratory epithelial cells can be partially explained by the methods used for lectin analysis as well as the type of lectin conjugate used. Antigen retrieval or unmasking did not become an established procedure in many laboratories until the mid 1990's and the earlier publications used paraffin embedded tissues without retrieval [3,8]. Later studies on pigs, primates and ducks also did not use retrieval procedures [9,17]. While the precise mechanism of retrieval still remains not precisely defined the general consensus is that heating or the use of enzymes serves to unmask antigenic sites that have become cross-linked through formalin fixation [18]. The unmasking of epitopes appears to extend to carbohydrate moieties as well as proteins. The increased detection of SAα2,6Gal through unmasking has also been recently shown in the liver [19].

Two isoforms of MAA have been recognized for many years and their binding profiles have been characterized. Since MAA1 bound to non-SAα2,3 glycans it has not been used as extensively by some researchers as MAA2. For instance, Shinya et al have recently demonstrated SAα2,3 Gal expression only in the lung but not the bronchus or upper respiratory tract [7]. Since they only used MAA2 lectin binding (Y. Kawaoka, personal communication) our MAA2 results are in accord with theirs. But our finding of MAA1 binding in the upper and lower respiratory tract does have implications for the possible distribution of receptors for avian influenza viruses including the currently circulating H5N1 viruses. The crucial question therefore is whether for the identification of susceptible binding sites for avian influenza viruses which are known to bind SAα2,3Gal, should researchers should just use MAA2 or should they also use MAA1? While both of these isoforms do identify the SAα2,3Gal ending they differ in their affinity for the inner fragments of the glycans, and these inner fragments are known to affect different virus binding [20]. To answer this we used glycan array data performed by the CFG for a known H5N1 avian influenza virus (Viet04) and also the affinity data for MAA1 (also known as MAL) [12]. As expected Viet 04 does have strong affinity for SAα2,3Gal terminated glycans and that MAL does identify SAα2,3Gal glycans but it also identifies non-SAα2,3 moieties and there is an overlap in the glycans that Viet04 and MAA1 have high affinity to [see Additional file 1]. Thus, there are H5N1 binding SAα2,3 terminated oligosaccharides that are detected with MAA1 (e.g. glycans 235–237), but these potential binding sites may not be detected if only MAA2 is used. We therefore believe that MAA1 should be used in conjunction with MAA2 in determining tissue distribution but do acknowledge that as it does detect non-SA terminated residues ancillary methods such as neuraminidase treatment of sections may be needed. Furthermore our findings that the MAA supplied by Roche and EY Laboratories primarily identifies MAA1 also indicates that these lectins should be used in conjunction with specific lectins from Vector labs to avoid misinterpretation of potential binding sites for SAα2,3 binding viruses. It is of note that our findings are applicable not only to influenza viruses but also to some parainfluenza viruses which also have strict SAα2,3Gal binding preference.

Because of this varied distribution of MAA1 and MAA2 throughout the respiratory tract, we hypothesised that this may shed new light on the distribution and binding of H5N1 viruses to the upper and lower respiratory tract. Using this information we therefore used ex-vivo cultures of the upper and lower respiratory tract and infected them with different H5N1, H1N1 and H3N2 viruses and found that contrary to previous suppositions, H5N1 viruses were able to replicate in the upper respiratory tract – a region which lacked MAA2 binding but had abundant MAA1 binding, thus indicating that the virus is perhaps binding to SAα2,3Galβ1,3/4GlcNac motifs or even to non-sialyated receptors [21].

Our results also indicated that the recent findings of Matrosovich and colleagues who found SAα2,3Gal in ciliated cells and SAα2,6Gal in the goblet cells of tracheobronchial cells cultured *in vitro *are only a partial representation of the true nature of sialic acid expression in the adult respiratory tract [2]. Their findings in the *in vitro *model with cultured tracheobronchial epithelium showed more similarity to the receptor profile seen in the respiratory tract of children. Therefore it is possible that the human tracheobronchial epithelial culture model is more representative of the respiratory tract of children rather than that of adults, and represents a developmentally earlier model of the human respiratory tract.

## Conclusion

In summary, we found that there was an overall increased detection of SAα2,3Gal in the respiratory tract when microwave unmasking is used and when a combination of different isoforms of MAA was used. We therefore advocate the routine use of this method for future investigations on the distribution of receptors for influenza viruses in the respiratory tract. We also urge attention to the exact isoform of MAA present in lectins supplied by different manufacturers [22]. These results imply a need for a re-evaluation of the findings reported in previous studies on the tissue distribution of SA receptor types. Furthermore, it appears that tissues of children have a greater expression of SAα2,3Gal than previously described, potentially conferring a greater susceptibility avian influenza viruses. Finally we were able to use the lectin histochemical findings to re-evaluate the tissue tropism of H5N1 infection of the respiratory tract and shed new light on the cells infected by this emerging virus by judicious used of both isoforms of MAA.

## Competing interests

The author(s) declare that they have no competing interests.

## Authors' contributions

Dr J Nicholls and Professor J S M Peiris designed the experiments and were responsible for primary analysis of tissues. Dr A J Bourne provided input to the CCAM cases from Australia. Dr H Chen and Y Guan assisted in the interpretation of results. All authors have read and approved the final manuscript.

## Supplementary Material

Additional file 1 Maackia amurensis 1 (MAA1), Sambucus nigra agglutinin (SNA) and H5N1 binding affinity in glycan array Description: Summary of glycan binding profiles of Maackia amurensis 1 (MAA1), Sambucus nigra agglutinin (SNA) and H5N1 influenza (A/Vietnam/1203/04) (Viet04) in the glycan array. Significant binding sugars are shown in column 2 with their glycan numbers according to Printed Array 2.1 shown in column 1. Strong affinity is **, weak affinity is * and no significant affinity is blank. Many SAα2,3Gal oligosaccharides (underlined) bind to both MAA1 as well as H5N1 (A/Vietnam/1203/4). Click here for file
