# Supplementary material for: Sialic acid receptor detection in the human respiratory tract: evidence for widespread distribution of potential binding sites for human and avian influenza viruses
Source: Respir Res. 2007 Oct 25;8(1):73. doi: 10.1186/1465-9921-8-73 (PMC2169242; doi:10.1186/1465-9921-8-73)
Supplement: Additional file 1 — Maackia amurensis 1 (MAA1), Sambucus nigra agglutinin (SNA) and H5N1 binding affinity in glycan array Description: Summary of glycan binding profiles of Maackia amurensis 1 (MAA1), Sambucus nigra agglutinin (SNA) and H5N1 influenza (A/Vietnam/1203/04) (Viet04) in the glycan array. Significant binding sugars are shown in column 2 with their glycan numbers according to Printed Array 2.1 shown in column 1. Strong affinity is **, weak affinity is * and no significant affinity is blank. Many SAα2,3Gal oligosaccharides (underlined) bind to both MAA1 as well as H5N1 (A/Vietnam/1203/4). [file 1465-9921-8-73-S1.pdf]

Glycan No	Glycan name	MAA1	SNA	Viet04
24	(Galβ1-4GlcNAcβ)$_2$-3,6-GalNAcα–Sp8	**		
27	[3OSO3][6OSO3]Galb1-4GlcNAcb-Sp0	*		
28	[3OSO3]Galb1-4Glcb-Sp8	**		
29	[3OSO3]Galβ1-4(6OSO3)Glcβ–Sp0	**		
30	[3OSO3]Galβ1-4(6OSO3)Glcβ–Sp8	**		
32	[3OSO3]Galβ1-3GalNAcα–Sp8	**		
33	[3OSO3]Galβ1-3GlcNAcβ–Sp8	**		
35	[3OSO3]Galb1-4[6OSO3]GlcNAcb-Sp8	**		
36	[3OSO3]Galβ1-4GlcNAcβ–Sp0	**		
37	[3OSO3]Galb1-4GlcNAcb-Sp8	**		
38	[3OSO3]Galβ–Sp8	**		
40	[4OSO3]Galb1-4GlcNAcb-Sp8			*
42	[6OSO3]Galβ1-4Glcβ–Sp0		*	
43	[6OSO3]Galβ1-4Glcβ–Sp8		*	
44	[6OSO3]Galβ1-4GlcNAcβ–Sp8	*	*	
45	[6OSO3]Galb1-4[6OSO3]Glcb-Sp8		*	
49	9NAcSAa2-6Galb1-4GlcNAcb-Sp8		**	
53	SAa2-3Galb1-4GlcNAcb1-2Mana1-3(SAa2-3Galb1-4GlcNAcb1-2Mana1-6)Manb1-4GlcNAcb1-4GlcNAcb-Gly		**	
54	SAa2-3Galb1-4GlcNAcb1-2Mana1-3(SAa2-3Galb1-4GlcNAcb1-2Mana1-6)Manb1-4GlcNAcb1-4GlcNAcb-Sp8		**	
67	Fucα1-2Galβ1-4(Fucα1-3)GlcNAcβ–Sp0	**		*
132	Galβ1-3GlcNAcβ1-3Galβ1-4Glcβ–Sp10	*		
143	Galb1-4GlcNAcb1-3(Galb1-4GlcNAcb1-6)GalNAca-Sp8	**		
144	Galβ1-4GlcNAcβ1-3GalNAcα–Sp8	**		
147	Galβ1-4GlcNAcβ1-3Galβ1-4GlcNAcβ–Sp0	*		
148	Galβ1-4GlcNAcβ1-3Galβ1-4Glcβ–Sp0	*		
149	Galβ1-4GlcNAcβ1-3Galβ1-4Glcβ–Sp8	*		
153	Galβ1-4GlcNAcβ–Sp8	*		
188	KDNα2-3Galβ1-4GlcNAcβ–Sp0	**		
202	SAa2-3Galb1-3GalNAca-Sp8			**
205	SAa2-8SAa2-8SAα2-3Galβ1-4Glcβ–Sp0	**		
212	NeuAca2-3(NeuAca2-3Galb1-3GalNAcb1-4)Galb1-4Glcb-Sp0			**
213	SAα2-3(SAα2-6)GalNAcα–Sp8			*
214	SAα2-3GalNAcα–Sp8			*
215	SAa2-3GalNAcb1-4GlcNAcb-Sp0			**
216	SAα2-3Galβ1-3(6OSO3)GlcNAc-Sp8	*		**
217	SAα2-3Galb1-3(Fucα1-4)GlcNAcβ–Sp8			**
218	NeuAca2-3Galb1-3(Fuca1-4)GlcNAcb1-3Galb1-4(Fuca1-3)GlcNAcb Sp0			**
219	SAα2-3Galβ1-3(SAα2-3Galβ1-4)GlcNAcβ-Sp8			**
220	Neu5Aca2-3Galb1-3[6OSO3]GalNAca-Sp8			**
221	SAα2-3Galβ1-3(SAα2-6)GalNAcα–Sp8			**
222	Neu5Aca2-3Galb-Sp8			**
223	NeuAca2-3Galb1-3GalNAcb1-3Gala1-4Galb1-4Glcb-Sp0			**
224	NeuAca2-3Galb1-3GlcNAcb1-3Galb1-4GlcNAcb-Sp0			**
225	SAα2-3Galβ1-3GlcNAcβ–Sp0			**
226	SAα2-3Galβ1-3GlcNAcβ–Sp8			**
227	SAa2-3Galb1-4[6OSO3]GlcNAcb-Sp8	**		**
228	SAα2-3Galβ1-4(Fucα1-3)(6OSO3)GlcNAcβ–Sp8			**
229	SAα2-3Galβ1-4(Fucα1-3)GlcNAcβ1-3Galβ1-4(Fucα1-3)GlcNAcβ1-3Galβ1-4(Fucα1-3)GlcNAcβ–Sp0			**
230	SAα2-3Galβ1-4(Fucα1-3)GlcNAcβ–Sp0			**
231	SAα2-3Galβ1-4(Fucα1-3)GlcNAcβ–Sp8			**
232	SAa2-3Galb1-4(Fuca1-3)GlcNAcb1-3Galb-Sp8			**
233	SAa2-3Galb1-4(Fuca1-3)GlcNAcb1-3Galb1-4GlcNAcb-Sp8			**
234	SAa2-3Galb1-4GlcNAcb1-3Galb1-4(Fuca1-3)GlcNAc-Sp0	*		
235	SAα2-3Galβ1-4GlcNAcβ1-3Galβ1-4GlcNAcβ1-3Galβ1-4GlcNAcβ–Sp0	**		**
236	SAα2-3Galβ1-4GlcNAcβ–Sp0	**		**
237	SAα2-3Galβ1-4GlcNAcβ–Sp8	*		**
238	SAa2-3Galb1-4GlcNAcb1-3Galb1-4GlcNAcb-Sp0	*		*
239	SAα2-3Galβ1-4Glcβ–Sp0	*		**
240	SAα2-3Galβ1-4Glcβ–Sp8			**
242	SAα2-6GalNAcα–Sp8		*	
243	SAa2-6GalNAcb1-4GlcNAcb-Sp0		**	
244	SAa2-6Galb1-4[6OSO3]GlcNAcb-Sp8		**	
245	SAα2-6Galβ1-4GlcNAcβ–Sp0		**	*
246	SAα2-6Galβ1-4GlcNAcβ–Sp8		**	
247	SAa2-6Galb1-4GlcNAcb1-3Galb1-4(Fuca1-3)GlcNAcb1-3Galb1-4(Fuca1-3)GlcNAcb-Sp0		**	
248	SAa2-6Galb1-4GlcNAcb1-3Galb1-4GlcNAcb-Sp0		**	
249	SAα2-6Galβ1-4Glcβ–Sp0	*	**	**
250	SAα2-6Galβ1-4Glcβ–Sp8		**	
251	SAα2-6Galβ–Sp8		**	
261	Neu5Gcα2-3Galβ1-4Glcβ–Sp0	**		
262	Neu5Gcα2-6GalNAcα–Sp0		*	
263	Neu5Gcα2-6Galβ1-4GlcNAcβ–Sp0		**	
